# Increased sensitivity in identifying language-related functional connectivity using jackknife resampling analyses

**DOI:** 10.1162/NETN.a.536

**Published:** 2026-04-22

**Authors:** Jinqing Liang, Divesh Thaploo, Adebiyi Sobitan, Kristen Wingert, Atsuko Kurosu, Stein Acker, Ahmad Shafiei, Ninet Sinaii, Nadia M. Biassou

**Affiliations:** The Integrative Neuroscience of Communication Research Unit, National Institute on Deafness and Other Communication Disorders, Bethesda, MD, USA; Biostatistics and Clinical Epidemiology Service, National Institutes of Health Clinical Center, Bethesda, MD, USA; Division of Neuroradiology, National Institutes of Health, Clinical Center Department of Radiology and Imaging Sciences, Bethesda, MD, USA

**Keywords:** Task-based fMRI, Static functional connectivity (traditional), Common connection, Jackknife analysis, Spearman, Pearson, Bonferroni correction

## Abstract

Functional connectivity (FC) analyses of task-based fMRI (tbfMRI) often rely on static correlation methods that average signal relationships over time. While widely used, these methods may miss transient but meaningful neural interactions. In this study, we investigated whether jackknife resampling—a technique that systematically omits one time point at a time—enhances sensitivity in detecting language-related FC networks during an auditory comprehension task. We analyzed surface-based FC networks in 172 healthy young adults from the Human Connectome Project. FC matrices were computed across 68 cortical regions of interest, and statistically significant edges were identified using Bonferroni correction. We compared FC networks derived from a traditional static correlation approach with those obtained using jackknife resampling, applying an edge consistency threshold to retain only the most stable connections across time points. The static method identified 75 significant language-related FCs. Jackknife-based analyses recovered all of these and revealed 24 additional connections or edges (eight left-hemispheric, five right-hemispheric, 11 interhemispheric; *p* < 0.001), including well-established language regions such as the middle temporal gyrus and posterior cingulate cortex. Jackknife resampling enhances detection of robust, task-relevant FCs, offering a promising alternative for modeling language networks and improving neurocomputational representations in both research and clinical settings.

## Author Summary

Functional connectivity analyses of task-based fMRI often rely on static correlations that may obscure transient but meaningful interactions. Here, we demonstrate that jackknife resampling—a leave-one-out approach across time points—enhances sensitivity in detecting language-related networks during an auditory comprehension task. Using data from 172 Human Connectome Project participants, jackknife-based analyses recovered all 75 connections identified by static methods and revealed 24 additional robust edges, including both intra- and interhemispheric links among canonical language regions. These findings highlight jackknife resampling as a promising alternative for improving the resolution and reliability of network representations in task-based fMRI studies.

## INTRODUCTION

Functional magnetic resonance imaging (fMRI) is a noninvasive neuroimaging method in which MRI images of brain structure are combined with BOLD signals, allowing us to measure the oxygen usage in different parts of the brain and thus measure brain activity on a per-region of interest (ROI) basis. This has been the basis of neuroactivational studies. The functions of certain brain regions largely rely on their interactions with other brain regions in a brain network. This is especially true for high-level cognitive tasks like language comprehension, which require integrated functioning of widely distributed brain regions (Démonet et al., 2005). For these reasons, there has been increasing interest in the interaction between ROIs, namely in the connectivity between discrete areas of functional neuro-activation (Shahhosseini & Miranda, 2022). Hence, studies of network correlations are of particular importance. Functional connectivity (FC) is the graphical representation of connectivity of discrete brain regions and is depicted as nodes and edges where the nodes represent the brain ROIs, and the edges represent the interaction between brain ROIs.

Pearson and Spearman rank correlation methods are commonly used to calculate the cross correlation between ROIs. However, in most scenarios, we cannot assume that the distribution of brain signal is normal. Therefore, the Spearman rank correlation is a preferred method in the calculation of FC (Lipkin et al., 2022). The jackknife method is a resampling method that boosts the number of samples by systematically leaving one observation out (Stahl & Gibbons, 2004). When comparing various dynamic FC methods, the jackknife method did perform on par with the sliding-window approach (Xie et al., 2019). There has been promising evidence regarding use of jackknife correlations (JCs) in understanding various emotional states (Ghahari et al., 2020). At the level of single-trial, correlations generated using the jackknife method outperforms traditional methods (Richter et al., 2015; Thompson et al., 2018). It can be combined with either Spearman or Pearson correlation, independent of the data distribution. This all-but-one resampling method results in a series of jackknife replications. For a sample *N* observation, *N* jackknife replications would be generated with each replication representing *N*−1 observations. However, the ability of traditional Spearman rank correlations, and JCs, to identify language related networks has not been elucidated to date. For this reason, we directly compared the language-related connectivity network maps generated using traditional (static) versus jackknife-Spearman and jackknife-Pearson correlations. Recent work in brain network analysis increasingly leverages representation learning and contrastive objectives to construct or embed graphs for clinical discrimination. Examples include structure–function fusing representation learning via adversarial decomposed variational autoencoders in mild cognitive impairment, and diffusion-based graph contrastive learning for disorder-sensitive network construction (Zong et al., 2024; Zuo et al., 2023). These approaches address how best to learn or embed brain graphs for downstream prediction. In contrast, the present study targets the upstream estimation problem in task fMRI: we evaluate whether jackknife resampling yields more stable, reproducible edges than static correlation, thereby providing a higher-confidence graph that could be used by subsequent learning methods. We hypothesize that the jackknife method will demonstrate relatively higher sensitivity in identifying language-related FC networks compared to static-based methodologies. Jackknife is a leave-one-out observation, which helps to identify how dependent the network connectivity is on specific data points, making it more robust against outliers and noise, which differs from Spearman, which does not assess variability due to time.

## METHODS

### Data Collection and Subjects

Task-based fMRI (tbfMRI) data from the Human Connectome Project (HCP) Young Adult database were obtained for healthy individuals who performed an auditory language comprehension task. All subjects participated in institutional review board (IRB)-approved research protocols performed at the respective institutions and made publicly available via permission granted through the HCP to the authors (N.B.). National Institutes of Health IRB analyst from the Office of Human Subject Research Protection deemed this research not to be new human subject’s data. Data from 262 healthy adults were queried, with 172 eligible for the study based on fMRI quality. They were matched for age, education, and handedness. Gender distribution was balanced (86 females [50%], 86 males [50%]) and racial demographics were diverse (57% White, 22% Asian/Hawaiian/Pacific Islander, and 14% Black or African American).

### Language Task and Image Acquisition

The language comprehension task was conducted as described by Binder et al. (2011) inside the scanner. Subjects listened to short stories and answered multiple-choice questions about the story’s topic. For example, after hearing a story about an eagle rescuing a man after the man had done the same for the eagle, participants might be asked, “Was the story about revenge or reciprocity?” In the original HCP paradigm, math problems served as a control condition; however, for the present study, only the story comprehension (language) condition was analyzed to ensure that the time series reflected language-specific neural activity.

We used the HCP Young Adult healthy volunteer dataset (S900 and S1200 releases), selecting subjects who had completed both resting-state and task-based fMRI, resulting in 172 participants. Two phase-encoding runs—right-to-left (RL) and left-to-right (LR)—were available per subject, each consisting of 147 volumes of story-only data after block separation and concatenation, yielding a total of 294 time points per subject for analysis. Handedness was assessed using the Edinburgh Handedness Inventory (EHI) (Barch et al., 2013; Ruck & Schoenemann, 2021), with EHI > 0 classified as right-handed and EHI < 0 classified as left-handed. Whole-brain gradient-echo echo-planar imaging (EPI) images were acquired on a 3 T Siemens Connectome Skyra MRI scanner (FOV: 208 × 180 mm; slice thickness: 2.0 mm isotropic voxels; multiband factor: 8; repetition time [TR]: 720 ms; TE: 33.1 ms; flip angle: 52°). Each run originally comprised six blocks (three story and three math), with each block lasting 30 s (21 volumes). For this analysis, only the story blocks per run were retained, concatenated within each run, and then combined across RL and LR runs. Structural T1- and T2-weighted images were collected for cortical reconstruction and segmentation. Functional data preprocessing, including distortion correction, motion correction, and censoring of volumes with framewise displacement (FD) > 0.3 mm, was performed using the Analysis of Functional Neuroimages (AFNI) toolbox (Cox, 1996; Cox & Hyde, 1997) and FreeSurfer (Fischl, 2012).

### Image Processing and Analysis

#### MRI processing.

Anatomical datasets including T1-MPRAGE and T2-SPACE were preprocessed by brightness normalization and skull stripping and then were registered with the Talairach-Tournoux brain atlas using an affine transformation. The FreeSurfer “recon-all” pipeline was used to generate 2D brain surfaces from MRI volumes. A brain mask file was first generated by FreeSurfer. The surface was created based on intensity gradients of the edges and interfaces between gray matter, white matter (WM), and pial. The reconstructed surfaces were then normalized to a template brain surface fsaverage (an average of 40 subjects). We then used the Desikan-Killiany atlas (Desikan et al., 2006) to parcellate the cortex into anatomically distinct regions. The normalized surfaces were mapped to the icosahedron to generate standard meshes for the group-level analysis, so that all the subjects will have the same node numbering of the brain surfaces.

#### fMRI processing.

The fMRI data was preprocessed using the AFNI toolbox. Slice-timing correction was performed to align all the slices to the beginning of the TR. The blip up/down nonlinear alignment was applied to EPI and then aligned the EPI to anatomical images. The WM intensity was uniformized across the brain volume, and the EPI masks were intersectioned with anatomical images for improved segmentation. Then, EPI images were then mapped to the standard surface mesh from the above MRI preprocessing section. After motion correction and slice-timing adjustment, we applied volume censoring at FD > 0.3 mm. Nuisance regression was performed using AFNI’s 3dTproject and included the six rigid-body motion parameters, their first temporal derivatives, and quadratic terms (12 motion regressors total), as well as mean WM and CSF signals (eight Region of Interest Principal Component regressors). A high-pass filter (∼0.008–0.009 Hz) was applied within the same model. No global signal regression was performed, and RETROspective Image-Based CORrection regressors were not available for these data. AFNI quality control indicated no frames exceeded this criterion, so no additional censoring was performed. JC was applied to the resulting continuous time series, generated for story blocks. The voxel-wise fMRI percent signal change data from the regression analysis were averaged within the ROIs from Desikan-Killiany atlas (Desikan et al., 2006).

### FC Network Analysis

In this study, we employed two complementary methods to investigate task-based FC:

a)  static functional connectivity (sFC), and
b)  JC approach.

### sFC (Traditional)

For each subject, task-based sFC was estimated by computing a 68 × 68 correlation matrix, where each element represented the functional connection (or “edge”) between two ROIs defined by the Desikan-Killiany atlas. In this study, the term “network” refers to the complete set of functional connections (edges) among the ROIs included in the analysis. An “edge” denotes a single, unique undirected connection between two ROIs. Throughout the Results section, “networks” denotes the entire collection of such edges that meet our inclusion criteria, whereas “edge” refers to individual ROI–ROI connection. The BOLD signal time series from each ROI was averaged, and pairwise correlations between ROIs were computed using both Pearson and Spearman rank correlation. The resulting correlation coefficients were transformed using Fisher’s Z transformation to normalize the distribution. To identify statistically significant edges within each subject, *p* values were computed using standard two-tailed tests for correlation under the null hypothesis of zero correlation. These calculations assume temporal independence; however, due to the structured nature of task-based designs, the effect of autocorrelation is reduced relative to resting-state fMRI (Afyouni et al., 2019; Lund et al., 2006; Woolrich et al., 2001). We applied a conservative Bonferroni correction across all ROI pairs (*α* = 0.05) to control false positives. This significance-based thresholding was used to retain robust and interpretable subject-level networks.

### JC Approach

To complement the static approach, we used the JC method to assess the temporal stability of FC estimates by systematically perturbing the time series. In this implementation, each iteration of JC systematically removed a single time point from the BOLD time series and recomputed the correlation matrix across all ROIs. This resulted in T jackknife iterations (where T is the number of time points), producing T correlation matrices per subject. Each matrix reflects the FC pattern based on a slightly perturbed dataset, allowing us to assess the stability and variability of each edge over time (Thompson et al., 2018). Following this, we applied an empirically defined edge retention criterion to determine which connections were consistently present across iterations. Specifically, for each ROI pair (i.e., each potential edge), we quantified how frequently a statistically significant correlation was observed across the T iterations. We evaluated JC stability thresholds from 70% to 90% in 10% increments across all subjects. Mean edge counts decreased slightly with higher retention (70%: 2291.99 ± 17.05; 80%: 2281.39 ± 5.32; 90%: 2278.08 ± 0.33), with only a 0.6% drop in density from 70% to 90%. The reduced variance at higher thresholds indicates more consistent edges across subjects. We selected 85% a bargain between stability stringency and network density, retaining most static FC edges while removing unstable connections (Supporting Information Figure S1). We did not compute per-time point *p* values rather on iterations itself; JC was used to quantify edge stability via leave-one-out resampling (85% retention), offering a within-context stability perspective that complements static FC.

### Statistical Methods

The Pearson and Spearman rank correlations and their respective *p* values, including Bonferroni correction for multiple correlations, were calculated using SciPy (v1.14.1). For each subject and task, edgewise *p* values were adjusted using Bonferroni across all 2,278 unique ROI pairs (*α* = 0.05, two-tailed). Significant edges satisfied *p*_adj_ < 0.05. At the group level, we assessed the proportion of jackknife iterations in which each edge exceeded the 85% retention threshold across participants. These retention proportions were tested against the null hypothesis of chance-level occurrence using a one-sample *t* test (two-tailed, *α* = 0.05). Only edges meeting both the within-subject Bonferroni criterion and the group-level retention significance test were considered reliably present. Demographic characteristics within the cohort were compared using Fisher’s exact tests for categorical data and the Cochran-Armitage trend test for ordinal data. The identified FCs by each method were compared using McNemar’s test for paired data, using the JC Pearson findings as the reference. All language–math comparisons are descriptive; no formal between-task inference was performed.

### FC Visualization

The FC edges were binarized and then visualized by BrainNet Viewer in Matlab (Xia et al., 2013).

### Hardware and Parallel Processing

The image processing and analysis was conducted on an Ubuntu Linux 18.04 workstation, Intel(R) Core(TM) i9-9960X CPU @ 3.10GHz, 128 GB RAM, and Nvidia Quadro RTX 5000 GPU. Parallel package was used to enable parallel processing for AFNI and FreeSurfer (Tange, 2021). For 68 ROIs and ∼300 timepoints, jackknife resampling involved ∼683,400 correlation computations per subject. On our workstation (Intel i9, 128 GB RAM), this required approximately 20–30 min per subject when executed serially, and 4–6 min with parallel processing (8–12 cores). In contrast, static FC computation required 1–2 min per subject.

## RESULTS

### Demographic Characteristics of Subjects

As shown in Table 1, subjects were aged 22–37 years old and equally distributed by gender. The racial distribution was 98 Whites (57%), 38 Asian/Hawaiian/Pacific Islander (22%), and 24 Black or African American (14%). They were predominantly (88%) right-handed. There were no differences in age between genders (*p* = 0.27) (or among racial groups (*p* = 0.20) or handedness (*p* = 0.16).

**Table 1.** Demographic characteristics of participants

**Characteristic**	***n* (%)**
**Age**	
22–25	61 (35.5)
26–30	69 (40.1)
31–37	42 (24.4)
**Race**	
Asian/Hawaiian/Pacific Islander	38 (22.1)
Black or African American	24 (14.0)
White	98 (57.0)
More than one race	6 (3.5)
Other/unknown	6 (3.5)
**Ethnicity**	
Hispanic/Latino	24 (14.0)
Not Hispanic/Latino	146 (84.9)
Unknown	2 (1.2)
**Gender**	
Female	86 (50)
Male	86 (50)
**Handedness**	
Left	19 (11.0)
Right	151 (87.8)
Neither	2 (1.2)

### Language-Related ROIs Identified by Traditional Static and Jackknife Correlations

**Figure 1.**
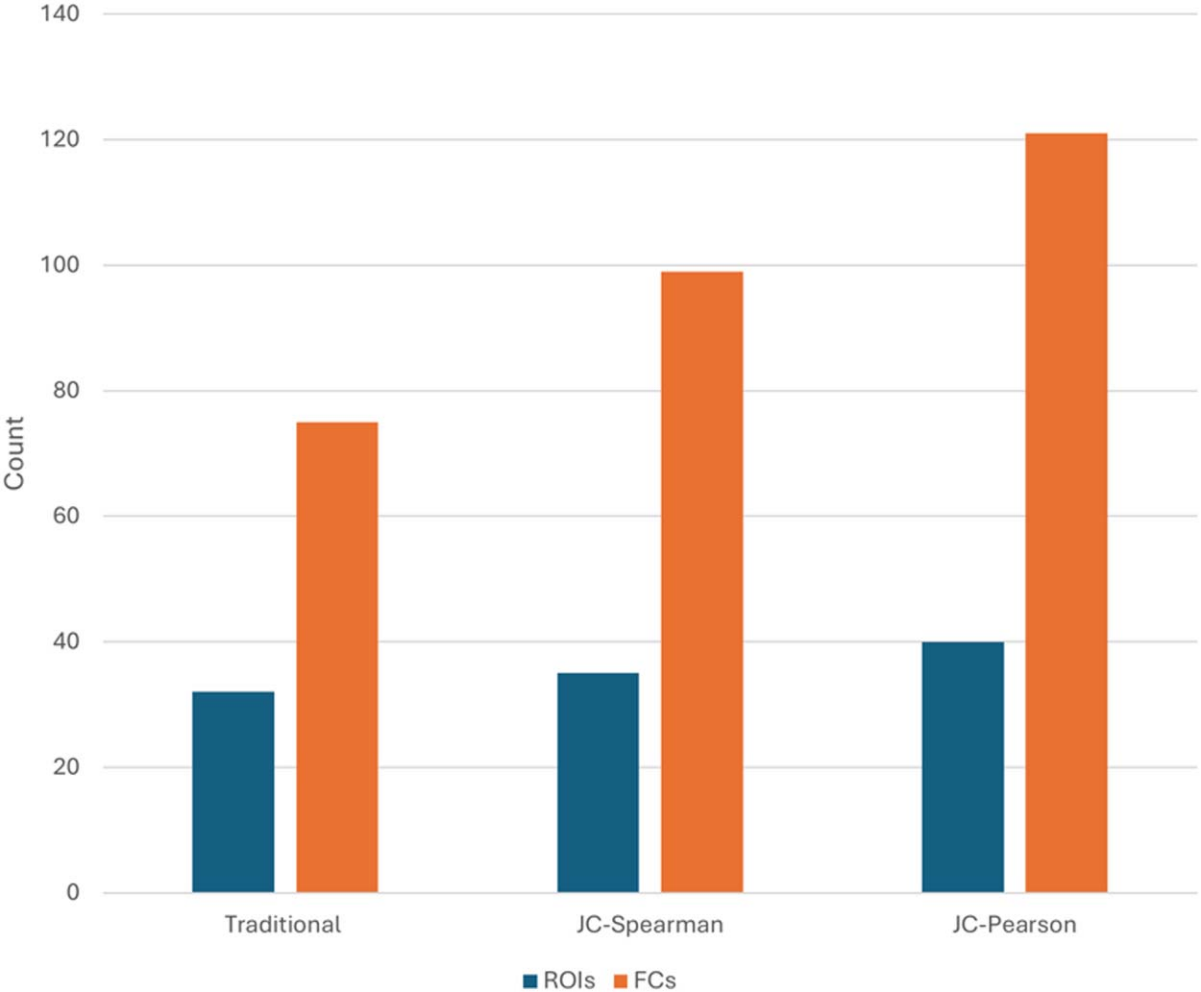
Number of FC networks identified by traditional static correlation and jackknife-Spearman and jackknife-Pearson correlation methods.

**Figure 2.**
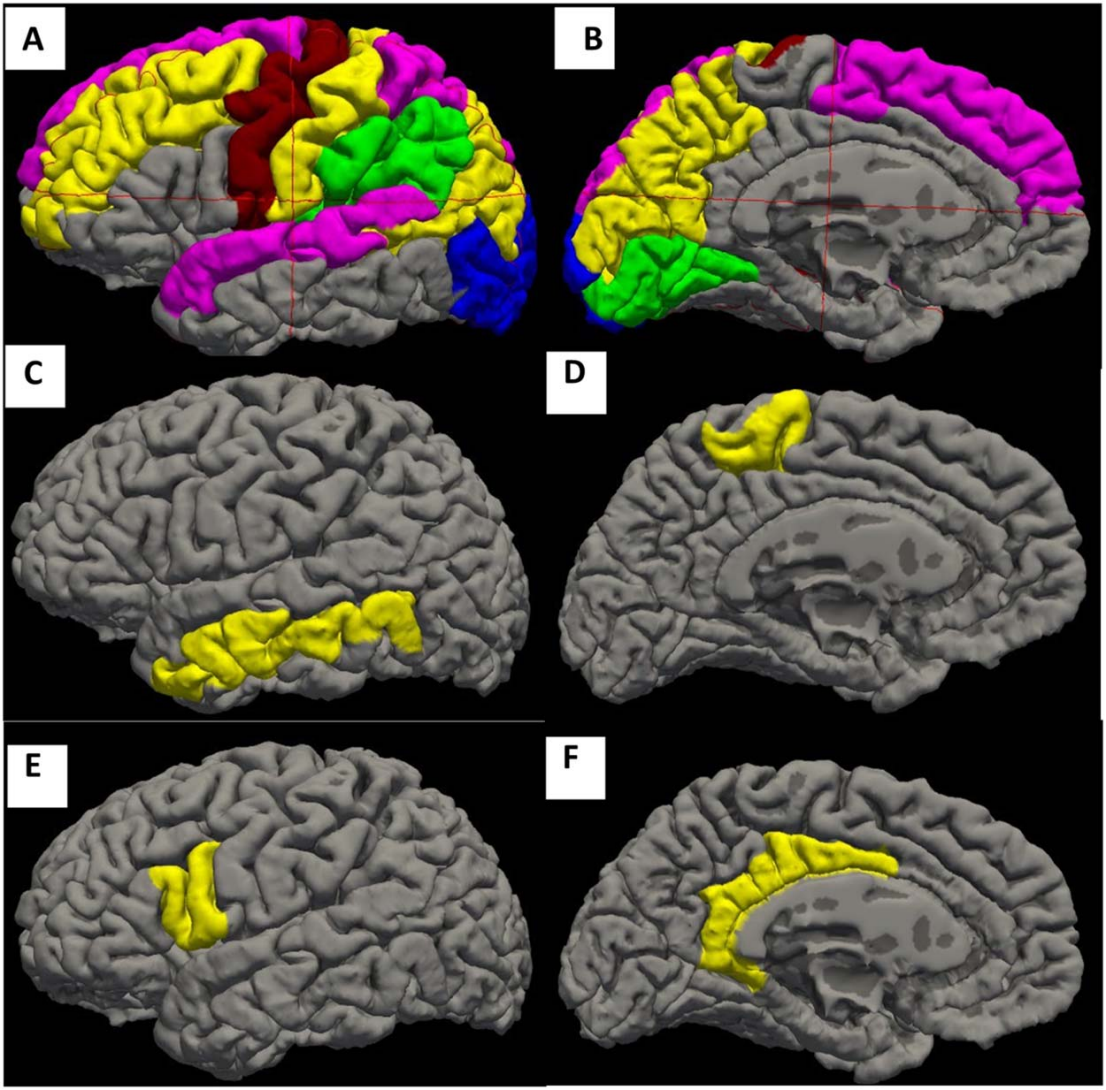
ROIs involved in FC in language tasks. The different colors represent different areas of the brain that demonstrate statistically significant FC network correlations across at least 85% of cohort subjects. A–B: The ROIs from the FCs identified by traditional static method. C–D: Represent the additional ROIs from the FCs identified by JC Spearman method compared to the traditional static method (ROI in C: middle temporal; ROI in D: paracentral). E–F: Represent the additional ROIs from the FCs identified by JC Pearson method compared to JC Spearman (ROI in E: pars opercularis; ROIs in F: isthmus cingulate, posterior cingulate). Only left hemisphere is shown here. The networks identified using jackknife are all associated with language networks previously described in the literature.

Traditional Static correlation analyses identified 75 edges from 16 ROIs of each hemisphere during the language tasks (Figure 1). These edges are from the brain regions that have been reported to be involved in language processing, understanding, and production (Bedny et al., 2011; Fedorenko et al., 2024) (Figure 2).

The JC Spearman method identified an additional three edges including middle temporal in the left and right hemispheres that are known to participate in language comprehension (Proverbio et al., 2009; Turken & Dronkers, 2011) and paracentral for language production (Arutiunian et al., 2023) Figure 2C–D, Table 2). The JC Pearson method identified another five ROIs compared to JC Spearman, which included the isthmus cingulate, posterior cingulate in left and right hemispheres, which are also known for language processing (Binder et al., 1997) and pars opercularis to produce speech (Lorca-Puls et al., 2021) (Figure 2E–F, Table 2).

**Table 2.** ROIs involved in language processing as identified by various methods

**ROI**	**Traditional**	**JC_Spearman**	**JC_Pearson**
lh_lingual	+	+	+
rh_pericalcarine	+	+	+
rh_superiorfrontal	+	+	+
lh_rostralmiddlefrontal	+	+	+
lh_transversetemporal	+	+	+
rh_lingual	+	+	+
rh_inferiorparietal	+	+	+
rh_superiortemporal	+	+	+
rh_bankssts	+	+	+
rh_supramarginal	+	+	+
lh_postcentral	+	+	+
lh_precuneus	+	+	+
rh_rostralmiddlefrontal	+	+	+
lh_caudalmiddlefrontal	+	+	+
lh_supramarginal	+	+	+
rh_precentral	+	+	+
lh_lateraloccipital	+	+	+
lh_superiorfrontal	+	+	+
lh_bankssts	+	+	+
rh_cuneus	+	+	+
rh_precuneus	+	+	+
lh_inferiorparietal	+	+	+
lh_pericalcarine	+	+	+
lh_superiorparietal	+	+	+
rh_postcentral	+	+	+
lh_cuneus	+	+	+
lh_precentral	+	+	+
lh_superiortemporal	+	+	+
rh_caudalmiddlefrontal	+	+	+
rh_transversetemporal	+	+	+
rh_superiorparietal	+	+	+
rh_lateraloccipital	+	+	+
rh_middletemporal	−	+	+
lh_middletemporal	−	+	+
lh_paracentral	−	+	+
lh_posteriorcingulate	−	−	+
rh_isthmuscingulate	−	−	+
lh_isthmuscingulate	−	−	+
rh_parsopercularis	−	−	+
lh_parsopercularis	−	−	+

+ refers to a ROI found and – refers to a ROI not found by the respective method.

### FCs Identified by Traditional Static and Jackknife Correlations

McNemar’s exact test revealed significant differences (*p* < 0.05) in the number of functional connections identified across the three approaches: traditional Spearman, jackknife-Spearman, and jackknife-Pearson. The jackknife-Pearson approach detected the greatest number of edges (121), significantly exceeding both traditional static (75 edges; *p* = 2.84 × 10^−14^) and jackknife-Spearman (99 edges; *p* = 4.77 × 10^−7^). As shown in Figure 1, traditional static correlation identified 75 FCs, whereas both jackknife approaches recovered 100% of these connections. Importantly, jackknife-Spearman and jackknife-Pearson analyses identified an additional 24 language-related networks (eight left-hemisphere, five right-hemisphere, 11 interhemispheric; *p* < 0.001) not detected by the static method. Furthermore, jackknife-Pearson identified 22 more language-related networks (11 left, five right, six interhemispheric; *p* < 0.001) than jackknife-Spearman (Supporting Information). Overall, jackknife-based correlations demonstrated greater sensitivity for detecting language-related FCs compared to traditional static analysis. Jackknife-Spearman revealed additional connectivity involving the superior parietal and precuneus in both hemispheres, while jackknife-Pearson further extended this detection to include supramarginal and superior frontal regions in the left hemisphere. As shown in Table 3, the traditional static correlation analyses identified 75 FCs. Both jackknife analyses with either Spearman or Pearson correlation identified 100% of FCs by traditional static method with additional edges identified by each, respectively (Table 3).

**Table 3.** The extra FC networks derived from jackknife-Spearman correlation and jackknife-Pearson correlation beyond what is identified via the traditional static analyses. The functional connectivity from jackknife method covers all the FCs from traditional static

**ROI1**	**ROI2**	**JC-Spearman**	**JC-Pearson**	**Tradition**
lh-bankssts	lh-superiortemporal	✓	✓	X
lh_cuneus	lh-superiorparietal	✓	✓	X
lh-inferiorparietal	lh-middletemporal	✓	✓	X
lh-inferiorparietal	lh-precuneus	✓	✓	X
lh-inferiorparietal	lh-rostralmiddlefrontal	X	✓	X
lh-middletemporal	lh-superiortemporal	X	✓	X
lh-parsopercularis	lh-supramarginal	X	✓	X
lh-rostralmiddlefrontal	lh-supramarginal	✓	✓	X
lh-superiorfrontal	lh-supramarginal	✓	✓	X
rh-inferiorparietal	rh-middletemporal	✓	✓	X
rh-postcentral	rh-supramarginal	✓	✓	X
rh-superiorfrontal	rh-supramarginal	X	✓	X
lh-inferiorparietal	rh-caudualmiddlefrontal	X	✓	X
lh-supramarginal	rh-precentral	X	✓	X
lh-supramarginal	rh-superiorparietal	X	✓	X
lh_transversetemporal	rh-superiortemporal	✓	✓	X

A ✓ indicates a newly identified edge by the corresponding network analysis, whereas X indicates the edge was not identified by that method.

## DISCUSSION

Investigating the neural basis of language comprehension has long been a central focus of cognitive neuroscience. Here, we demonstrate that applying jackknife resampling to task-based fMRI significantly enhances the detection of language-related FC networks. Compared to traditional static correlation methods, jackknife-derived FCs were more sensitive in capturing consistent, task-relevant edges, including known language-associated regions that were otherwise undetected. The newly identified edges were not arbitrary but align with connections previously reported in the literature as being involved in language processing, thereby supporting the validity of these methods (Towle et al., 2008). This improved sensitivity likely stems from the jackknife method’s ability to probe the stability of network connections by systematically evaluating their robustness across time. By recomputing FC after removing each time point, jackknife resampling functions as a natural variability test, reducing the influence of outliers and improving the reproducibility of identified edges. This addresses one of the core challenges in network neuroscience—estimating reliable, meaningful graphs from noisy neuroimaging data.

While the possibility that additional networks were identified by chance must be considered, we addressed this through conservative Bonferroni correction and an empirically derived edge retention threshold (85% occurrence). The fact that many of the additional connections were previously implicated in semantic, syntactic, and attentional processes—such as the middle temporal gyrus and posterior cingulate—supports the neurobiological plausibility of our findings. Moreover, the additional networks identified via jackknife methodologies are known to be language-specific networks (see Figure 2). Specifically, it correctly identified the left middle temporal gyrus, which has been identified as being important in the ventral pathway structures of language processing in the human brain, namely for syntactic/semantic processing and the left pars opercularis, which is posited to be part of the dorsal pathway and particularly important in phonological processing/speech planning (Hickok & Poeppel, 2004; Saur et al., 2008) (see Figure 2B). Jackknife-Pearson methodology also identified language-related cortices, that is, associative cortices that subserve processes, such as attention, that support the processing of language specific. For example, it identified the isthmus cingulate and posterior cingulate that have been reported as playing critical roles in regulating attention (Leech & Sharp, 2014) (see Figure 2c).

Although we report the benefits of jackknife-based FC estimation in a static context, this method may also serve as a powerful tool for exploring dynamic connectivity. Unlike traditional sliding-window approaches, which rely on arbitrary time segmentation, jackknife resampling provides an edge-centric, time-resolved measure of stability. This feature makes it particularly attractive for modeling dynamic neural processes across tasks or clinical states, where time-resolved network reliability is critical (Tian & Poeppel, 2013). The large variability of FC was observed in the hetero-modal associative cortices including some of the brain regions for language comprehension while minimal variability of FC was found in the unimodal sensory and motor cortices (Mueller et al., 2013). Frost and Goebel also reported that language areas demonstrated large variability among individual subjects (Frost & Goebel, 2012). We think that the use of such methodologies may result in the development of more robust neurocomputational modeling of language-related changes in FC networks secondary to central nervous system injury as measured by tbfMRI (Acker et al., 2024). An additional advantage is the method’s nonparametric nature, allowing its use without assumptions about signal distribution. Although there is not a lot of literature out there, a recent study pointed out its significance (Kuo & Liou, 2022). While computational demands are nontrivial, especially in high-resolution datasets, the method’s ability to highlight robust, reproducible networks suggests strong potential for future applications. These may include longitudinal tracking of neural changes, individualized network fingerprinting, and improved neurocomputational modeling of cognitive function and dysfunction. The JC method estimates the stability of ROI–ROI correlations by iteratively removing one time point from the fMRI time series and recalculating connectivity. While agnostic to explicit task timing, its interpretability in task fMRI depends on design: event-related paradigms may allow JC to capture finer temporal fluctuations, whereas in block designs, as used here, JC primarily reflects stable connectivity patterns across the run. JC performance is influenced by time series length, with longer runs yielding more stable estimates and shorter runs more susceptible to variability due to the proportionally greater impact of omitting a single time point; in our case, the relatively long HCP runs mitigated this concern. Conceptually, JC is related to approaches such as beta-series correlations or contrasts of condition-specific general linear mode betas but differs in being design-agnostic and not requiring explicit condition modeling, making it a complementary tool for examining connectivity stability in contexts where condition-specific modeling is less feasible.

However, several limitations should be noted. First, while jackknife resampling improves sensitivity, it does not eliminate the potential for spurious correlations, especially in the presence of residual motion artifacts or nonstationary signal fluctuations. The 85% setting is an empirically guided choice on this dataset; comprehensive optimization and formal threshold selection will be addressed in future work. Third, our analysis was confined to group-level trends and did not assess the method’s utility for individualized predictions, which will be essential for future translational applications. Finally, the method’s computational burden may limit its feasibility in studies with large datasets or real-time applications, highlighting the need for optimization or parallelization strategies.

To our knowledge, this is the first study to apply jackknife resampling to language task fMRI data for the purpose of FC estimation. The observed increase in sensitivity and reproducibility positions this method as a valuable addition to the network neuroscience toolkit, particularly in contexts where high precision and robustness are essential.

### Ethics

All subjects participated in IRB-approved research protocols performed at the respective institutions and the existing datasets made publicly available via HCP with permission granted to the senior author. NIH IRB analyst from Office of Human Subject Research Protection deemed this research not to be new human subject’s data.

## Acknowledgments

We thank Dr(s). Barry Horwitz, Senior Investigator Emeritus and Nusrat Rabbee, Chief of the Dept of Biostatistics and Epidemiology, NIH Clinical Center National Institutes of Health for their extremely helpful comments on early drafts of this manuscript.

## Supporting Information

Supporting information for this article is available at https://doi.org/10.1162/NETN.a.536.

## Author Contributions

Jinqing Liang: Formal analysis; Software; Visualization; Writing – original draft. Divesh Thaploo: Formal analysis; Software; Validation; Writing – review & editing. Adebiyi Sobitan: Supervision; Validation. Kristen Wingert: Writing – review & editing. Atsuko Kurosu: Writing – review & editing. Stein Acker: Writing – review & editing. Ahmad Shafiei: Writing – review & editing. Ninet Sinaii: Formal analysis; Methodology; Software; Supervision; Validation; Writing – review & editing. Nadia M. Biassou: Conceptualization; Formal analysis; Funding acquisition; Investigation; Methodology; Project administration; Resources; Supervision; Validation; Writing – original draft; Writing – review & editing.

## Funding Information

Nadia M. Biassou, NIH Clinical Center (https://dx.doi.org/10.13039/100000098), Award ID: ZIA CL090077. Nadia M. Biassou, National Institute on Deafness and Other Communication Disorders (https://dx.doi.org/10.13039/100000055), Award ID: Intramural Research Program Funding.

## Data and Code Availability

The data used in this paper are freely available from https://db.humanconnectome.org/ (requires free registration). All code for this paper is available at Github: THINC_LABS/NN_paper at master dthaploo/THINC_LABS.

## Supplementary Material



## TECHNICAL TERMS

Regions of interest (ROIs): Predefined brain areas chosen for connectivity analysis, often based on anatomical or functional atlases.
Functional connectivity (FC): Statistical dependencies between spatially distinct brain regions, reflecting how neural activity co-varies over time.
Jackknife resampling: A leave-one-out statistical method systematically omitting single time points to test stability and robustness of estimates.
Static correlation: Averaging connectivity measures across the entire scan, ignoring time-varying fluctuations in neural interactions.
Task-based fMRI (tbfMRI): Brain imaging during specific tasks to measure task-evoked neural activity and connectivity patterns.
Human Connectome Project (HCP): Large-scale neuroimaging dataset providing high-quality structural and functional brain data from healthy individuals.
Auditory comprehension task: Language-related fMRI paradigm where participants process spoken sentences to engage comprehension networks.
Bonferroni correction: A multiple-comparison statistical adjustment that lowers significance thresholds to reduce false positives.
Edge consistency threshold: A stability filter retaining only network connections consistently observed across multiple resampled datasets.
Interhemispheric connectivity: Functional links between homologous or complementary brain regions across the left and right hemispheres.
