# Supplementary material for: Increased sensitivity in identifying language-related functional connectivity using jackknife resampling analyses
Source: Netw Neurosci. 2026 Apr 22;10(2):267–80. doi: 10.1162/NETN.a.536 (PMC13108337; doi:10.1162/NETN.a.536)
Supplement: Supplementary file 1 [file netn-10-2-267-s001.pdf]

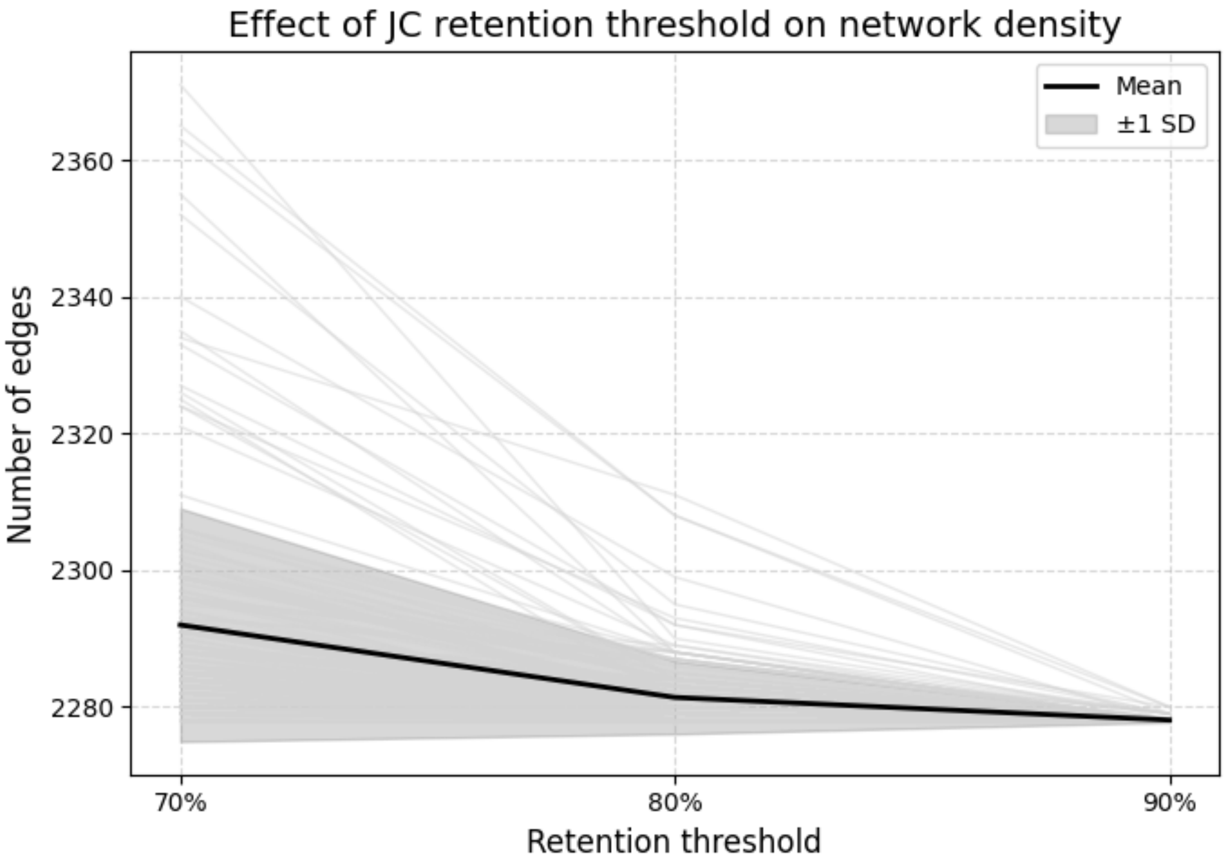

Figure 1 Effect of Jackknife correlation (JC) retention threshold on network density. Increasing the threshold from 70% to 90% led to a gradual decrease in the number of retained edges, with the mean shown in black and shaded area representing ±1 SD. Based on this evaluation, we selected an 85% retention threshold as it balanced network density preservation with improved robustness against spurious connections.

Biological validity of the 24 (Spearman) / 46 (Pearson) "new" edges

We performed McNemar exact test to assess significant differences in edges identified by traditional Spearman, Jackknife Spearman, and Jackknife Pearson approaches.

Exact McNemar Test (with central confidence intervals)
Functional Connections (edges)

		Jackknife Pearson			
		Present	Absent	Total	p-value (conditional)
Traditional spearman	Present	75	0	75	2.84E-14
	Absent	46	0	46	
	Total	121	0		

		Jackknife Spearman			
		Present	Absent	Total	p-value (conditional)
Traditional spearman	Present	75	0	75	1.19E-07
	Absent	24	0	24	
	Total	99	0		

		Jackknife Spearman			
		Present	Absent	Total	p-value (conditional)
Jackknife Pearson	Present	99	22	121	4.77E-07
	Absent	0	0	0	
	Total	99	22		

McNemar's exact test (conditional) showed significant differences ($p < 0.05$) in the number of edges identified by all three approaches: traditional Spearman, Jackknife Spearman, and Jackknife Pearson approaches. The Jackknife Pearson approach identified 121 edges which is significantly higher than edges identified by Traditional Spearman (75 edges, p = 2.84e-14) and Jackknife Spearman (99 edges, p = 4.77e-07).
